# Can Blood Gene Expression Predict Which Patients with Multiple Sclerosis Will Respond to Interferon?

**DOI:** 10.1371/journal.pmed.0020033

**Published:** 2005-02-22

**Authors:** Naftali Kaminski, Anat Achiron

## Abstract

Gene expression patterns from peripheral blood cells may be useful as biomarkers for monitoring MS progression and response to therapy, argue Kaminski and Achiron

Despite the significant progress in increasing our understanding of the immune mechanisms of multiple sclerosis (MS), in improving clinical classification and brain imaging, and in developing new treatments, the factors that determine the course of the disease are mostly unknown [1]. Currently, it is nearly impossible to predict the course of MS, its severity in terms of disability progression, or when a relapse will happen.

The most commonly used disease-modifying therapies are interferon β (IFNβ) [2] and glatiramer acetate [2,3]. Despite initial excitement, these therapies have beneficial effects in some, but not all, patients [2,3]. Because of the potential favorable effects of these therapies, it has been suggested that they should be initiated as early as possible to maximize neuroprotection [4]. Additionally, it has been recommended that patients should be monitored closely to determine whether and when it is necessary to modify treatment in order to maximize the benefit [5]. The recommended monitoring is based on annual rate of relapses, neurological deterioration, and evidence of disease activity on brain magnetic resonance imaging scans. However, given the destructive nature of the disease, if we rely solely on clinical or radiological manifestations (such as a relapse or a new lesion on a scan) to determine a patient's response to therapy, we will probably be responding too late.

## Gene Expression Patterns in Affected Organs

The diagnosis and management of disease could be transformed thanks to the completion of the human genome project, the availability of sequence information for nearly every gene, and the advent of novel high throughput technologies (microarrays—see Glossary) that allow parallel profiling of thousands of genes. By definition, nearly every aspect of a disease phenotype should be represented in gene expression signatures of multiple genes in the affected organ. Indeed, studies that analyze affected tissues (mostly in cancer) clearly show that it is possible to predict prognosis, to identify new classes of diseases, and potentially to determine response to therapy [6,7,8].

**Glossary**

**cDNA arrays:** Microarrays in which the gene detectors are pieces of cDNA.

**Cross-validation:** A method by which an available sample is split into learning and testing sets to test classifiers.

**Gene expression signature:** Statistically significant changes in the expression of multiple genes that characterize (classify) a biological state.

**Glatiramer acetate:** A synthetic protein made of four amino acids found in myelin. It is used as an immunomodulator drug in treating MS.

**IFNβ:** A cytokine that is secreted from fibroblasts in response to stimulation by a live or inactivated virus or by double-stranded RNA. It is used as an immunomodulator drug in treating MS.

**Microarray:** A technology that allows the simultaneous profiling of the expression of thousands of genes (even whole genomes). Multiple gene detectors (oligonucleotides or cDNAs) are deposited on a slide that is hybridized with fluorescently labeled samples.

**PCR (polymerase chain reaction):** The exponential amplification of a DNA fragment using repeated activation of a heat-stable DNA polymerase.

**Real-time PCR (also called one-step kinetic RT-PCR):** A method in which the quantitation of the products of PCR is made by measuring fluorescent emission. It is used for accurate quantitation of mRNA.

**RT-PCR (reverse transcription–polymerase chain reaction):** PCR that is performed on cDNA generated from RNA. It is used for mRNA detection and quantitation.

**Supervised classification:** A process in which classifiers are learned from user-defined groups (classes).

**Unsupervised classification:** A process in which classifiers are learned without user-defined groups (classes), i.e., without a predefined training set.

In diseases that do not require tissue resection for diagnosis or therapy, it is rare to obtain tissues for analysis. This problem is even more pronounced in diseases like MS, in which the target organ is the very inaccessible brain and spinal cord. Despite these limitations, several groups used microarrays to analyze brain tissues obtained posthumously from patients who had MS and identified genes that characterized either acute or chronic lesions [9,10,11]. However, although these studies identified some potential genes that may be involved in the local pathogenesis of the disease, they did not produce any information that could be used for identifying biomarkers associated with disease activity.

## Diagnostic Peripheral Blood Mononuclear Cell Gene Expression Signatures

In MS, looking for markers of disease activity in the much more accessible peripheral blood does not require a significant leap of faith. MS is an autoimmune disease, and it is possible that some of the cells involved in the pathogenesis of the disease will be found in the bloodstream. Abnormal T cell populations have repeatedly been observed in the peripheral blood of patients with MS [12,13,14]. While these results supported looking at the easily accessible peripheral blood mononuclear cells (PBMCs) for potential markers that reflect the disease, some doubts persisted. These revolved around two very strong arguments. The first argument was that if the signal comes from a minority of the cells within the bloodstream it will be too low to be detected. The second was that interpersonal variability, added to the inherent noisy nature of gene expression data, will make the data impossible to reproduce.

Fortunately, recent observations suggest that these doubts are unfounded. Bomprezzi et al. [15] determined that gene expression patterns can distinguish patients with MS from controls and suggested that at least some of the differences identified were derived from activated T cells. Achiron et al. [16] analyzed the expression of 12,000 genes in patients with relapsing–remitting MS. Gene expression patterns clearly distinguished patients with MS from controls as well as relapse from remission. Mandel et al. [17] compared patients with systemic lupus erythematosus and MS, and identified a common autoimmunity signature as well as disease-specific gene expression signatures. Interestingly, similar findings were recently described for pulmonary arterial hypertension [18].

## Could PBMC Gene Expression Signatures Be Used for Predicting Response to Therapy?

Weinstock-Guttman et al. [19] analyzed the acute transcriptional response of 4,000 genes in peripheral blood lymphocytes to IFNβ. They identified increases in known interferon-inducible genes, and in genes involved in antiviral activity and interferon signaling. Using complementary DNA (cDNA) arrays, Sturzebecher et al. [20] identified gene expression signatures that distinguished IFNβ responders from nonresponders.

**Figure 1:**
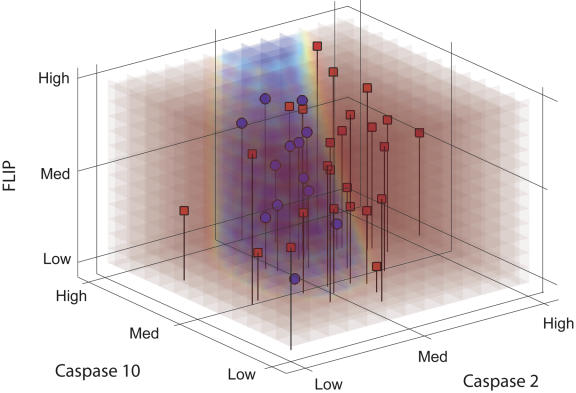
Expression Levels of Three Genes in Patients Who Responded (Red) and Who Did Not Respond (Blue) to IFNβ (Source: [21])

And now, in a new study published in last month's *PLoS Biology*, Baranzini et al. [21] provide compelling evidence that these PBMC gene expression signatures can be used to predict response to therapy (Figure 1). They studied the expression of 70 genes selected for their presumed biological function in 52 patients with MS, followed up for at least two years after initiation of IFNβ therapy. Instead of using microarrays that carry probes for thousands of genes, they chose to use real-time PCR. This method is highly sensitive, specific, and reproducible across different laboratories. It is often used to verify microarray findings. Baranzini et al. identified MX1 (interferon-inducible protein p78), a known interferon-inducible gene, as the marker of treatment with IFNβ. They did not find overall differences between responders and nonresponders, but they did, using supervised classification methods, identify triplets of genes that distinguish IFNβ responders and nonresponders.

Interestingly, individual and pairs of genes did not perform that well, and all three genes in a triplet were required for the highest accuracy (about 80%–90%). The minimal combinatorial number of genes that contains the most predictive information is not available since combinations of more than three genes were not performed. Although the results were not tested on an independent dataset, as is frequently requested [22], the authors applied an array of cross-validation strategies that convincingly suggested that the identified predictive signal was robust.

## Implications of the Study

What could Baranzini and colleagues' findings mean? Clearly, the most obvious conclusion is that the lack of response did not result from the deactivation of IFNβ. The effect of IFNβ on MX1, IFNAr1, and STAT2 was observed for two years in all patients, suggesting that the response did not depend on IFNβ bioavailability. Considering that PBMCs represent an admixture of multiple cell types, the most plausible explanation is a simple lack of shift in subcellular populations.

However, the importance of Baranzini and colleagues' study lies not in its mechanistic insights, but in its clinical relevance. The careful design of the experiment, the use of reproducible real-time PCR instead of microarrays, the meticulous analysis, and the previous observations [15,16,17,19,20] support the notion that PBMCs express clinically relevant gene expression signatures in MS and probably in other organ-confined diseases. To further prove this notion will require a significant investment in large studies that prospectively test the utility of these signatures in guiding the management of MS. Only when direct evidence shows that therapy guided by markers expressed in PBMCs improves patient outcome will PBMC gene expression patterns take their place as biomarkers at the center stage of monitoring MS progression and response to therapy.

## Abbreviations

cDNA: complementary DNA
IFNβ: interferon β
MS: multiple sclerosis
PBMC: peripheral blood mononuclear cell
